# Largest Cotton Planter in the World

**Published:** 1882-11

**Authors:** 


### LARGEST COTTON PLANTER IN THE WORLD.

IN a letter from Atlanta, Ga., a correspondent of a New York paper says: It is by brains, and not by the mere vastness of his farming operations, that Mr. Edward Richardson, of Mississippi, the greatest cotton-raiser in the world, has amassed his immense fortune, now estimated at from $15,000,000 to $20,000,000. The means by which Mr. Richardson has achieved his phenomenal success as a planter are worthy a moment's study for the lessons they convey. His business is a comprehensive one, including everything relating to cotton. He not only raises cotton, but gins, spins and weaves it, is a large dealer, and has oil mills as well. He was clear-sighted enough to perceive that there is a special profit in each process and operation through which cotton passes from the field to the consumer of cotton goods, and he had the capital and ability to organize a business which makes all these profits his own. He owns some 52,000 acres of land, and last year raised over 12,000 bales of cotton—a greater number than the Khedive of Egypt, who is the next largest cotton-raiser in the world. Mr. Richardson is not a "high" farmer, a bale to three acres being the average production of his land, which is largely tilled by tenants on the share system. The 36,000 pounds of seed cotton which he annually gets from his land are ginned by his own gins—which do public ginning also—and pressed, baled, and compressed, so much as is shipped as raw material, on his own plantation. The seed, which is ordinarily not worth more than $6 a ton, and is to a great extent wasted by other planters, is ground and pressed for the oil. The hulls are used for fuel in this process, and the ashes sold and used for fertilizers. From a ton of seed he obtains thirty-five gallons of oil, worth 35 cents a gallon—$12.25. The cake remaining after the oil is pressed out is worth rather more for feed or as a fertilizer than the seed itself, selling readily for home use or shipment to England at $6 to $7 a ton. Each ton of cotton-seed, therefore, nets rather more than $20—the bulk used as fuel being taken into account. Mr. Richardson's mills at Corinth receive and manufacture a large part of his crop, and another profit is added from the sales of yarns and sheetings, drillings, cottonades, etc., a profit which is considerably enhanced by the elimination of shipping charges, insurance, broker's commission, and other tolls levied on cotton shipped to distant mills.

---

COMMUNISM possesses a language which every people can understand. Its elements are hunger, envy, death.—*Heinrich Heine.*

